# Diurnal and Seasonal Occurrence of Febrile Seizures

**DOI:** 10.15844/pedneurbriefs-29-4-4

**Published:** 2015-04

**Authors:** John J. Millichap, J. Gordon Millichap

**Affiliations:** 1 Division of Neurology, Ann & Robert H. Lurie Children's Hospital of Chicago, Chicago, IL; 2 Departments of Pediatrics and Neurology, Northwestern University Feinberg School of Medicine, Chicago, IL

**Keywords:** Children, Daylight, Diurnal Variation, Febrile Seizure, Melatonin, Seasonal Variation

## Abstract

Investigators from University of Oulu, Finland, evaluated the diurnal and seasonal occurrence of the first febrile seizures (FS) in 461 children in a population-based study of 1522 children.

Investigators from University of Oulu, Finland, evaluated the diurnal and seasonal occurrence of the first febrile seizures (FS) in 461 children in a population-based study of 1522 children. The first FS occurred most often in the evening, peaking between 6 and 10 PM (31%), and least often at night, in the early morning hours between 2 and 6 AM (8%) (P < 0.001). This diurnal pattern was repeated in different seasons according to the variance in daylight duration. FS occurred irregularly throughout the year, most frequently in the winter, concurrently with the febrile episodes, and least frequently in the summer. The diurnal and seasonal variations in the occurrence of FS did not follow the amount of daylight throughout the day or year or variation in the secretion of melatonin. Rather, FS are strongly associated with episodes of fever, infections, and elevated body temperature. The maximum temperature of the fever in patients with FS (mean 39.7 °C, range 37.6-42.0 °C) was similar throughout the year. [1]

COMMENTARY. The seasonal and diurnal variations in the occurrence of febrile seizures (FS) observed in children in Finland [1] are reported also in Japanese [2] and in US children [3]. The frequency of FS is highest in the winter and lowest in summer months in all 3 geographic areas, and approximately 5 times greater in the evening than in early morning [2]. FS propensity is not related to circadian rhythm or amount of daylight but rather to the elevation in temperature. The maximum occurrence of FS at 4.00 pm, and the minimum, at 4 am, is similar to the pattern of human body temperature [2]. The elevation in temperature is usually associated with a viral infection, especially human herpesvirus-6 [4].

Several factors may be involved in the cause of FS, including age dependency (3 months to 5 years; maximum 12-18 months), genetic susceptibility (family history, autosomal dominant with reduced penetrance, or multifactorial), cytokine and immune response to infection, fever and height of body temperature. The maximum height of a fever, not the rate of rise, is the main determinant of risk of FSs, a finding demonstrated in animals [5] and confirmed in clinical studies [6, 7]. In a study of 110 children with febrile seizures, the difference between temperature recordings associated with and those without FS was highly significant (P < 0.001). Seizures occurred when the degree of fever reached or surpassed the threshold convulsive temperature for each patient [6].
